# Detection of high-risk Human Papillomavirus in prostate cancer from a UK based population

**DOI:** 10.1038/s41598-023-34734-3

**Published:** 2023-05-10

**Authors:** M. Yahya Ahmed, Nadia Aziz Salman, Sarbjinder Sandhu, M. Okan Cakir, Alan M. Seddon, Christian Kuehne, G. Hossein Ashrafi

**Affiliations:** 1 School of Life Science, Pharmacy and Chemistry, Kingston University London, London, KT1 2EE UK; 2 Department of Urology and Surgery, Kingston Hospital, Kingston upon Thames, London, KT2 7QB UK; 3 CEO Valdospan GmbH, Technopark 1D, 3430 Tulln an der Donau, Austria

**Keywords:** Prostate cancer, Oncology, Urology

## Abstract

Human papillomavirus (HPV) infection is one of the sexually transmitted diseases which have been implicated in the etiology of multiple cancers. To date, several studies have been conducted to evaluate the incidence of high-risk (HR) HPV in prostate cancer (PCa) which have generated widely conflicting data. Hence, this leaves a lack of awareness on the causal role of persistent HPV infection in the development of PCa. Although this has been investigated in a handful of countries, to the best of our knowledge, no prior studies have been conducted in the UK. In this study, polymerase chain reaction (PCR) and Sanger sequencing were implemented to analyze a total of 49 fresh prostate specimens (35 benign and 14 malignant specimens) for the presence of viral DNA of 12 HR-HPV types. Data obtained confirmed the presence of HR-HPV in 32.7% of analyzed benign and malignant prostate tissues with HPV 35 being identified as the most frequent type. Moreover, HR-HPV positivity rate was found to be higher in abnormal prostate tissues (adenocarcinoma and benign with prostatitis) compared those with normal prostate condition. Using immunohistochemistry, we have confirmed the expression of HPV E7 protein in prostate tissues positive for HPV DNA. This observation, the first reported from a UK population, suggests that the presence of HPV in prostate tissue is likely to be a related factor in the progression of certain cases of prostate cancer.

## Introduction

Prostate cancer (PCa) is the most common diagnosed cancer among men in the UK and second most prevalent type of cancer in men worldwide after lung cancer^1^. An estimate of 48 600 new PCa cases with more than 12,000 deaths are recorded in the UK annually^2^. Multiple risk factors have been associated with the development of PCa, such as, genetic factors, ethnic origin, testosterone level, chronic inflammation and infection of the prostate^3^ although, the initiating causal agents are yet to be identified. This has stimulated this search to identify new etiological factors associated with this neoplasia^4,5^.

Infectious agents have been implicated as either direct carcinogens or promoters. In particular, high-risk Human Papillomaviruses (HR-HPVs) have been identified as carcinogenic agents in humans. HPVs are small, non-enveloped viruses, from a large family of double stranded circular DNA viruses that may infect epithelial surfaces (skin, genital) through sexual/skin-to-skin contact and cause hyper-proliferative lesions (known as warts or papillomas). To date, over 200 HPV types have been discovered and classified as “low-risk” or “high-risk” types depending on their association with the development of neoplasms. The low-risk HPV types cause benign lesions whereas the high-risk types are associated with carcinogenesis^6,7^.

Long term viral persistence, which requires evasion of the body’s immune attacks and clearance is essential for malignancy. It has previously been shown that, like many other viruses, HPVs have several ways of subverting an effective immune response that may contribute to delay in, or lack of clearance of HPV infections^7–9^. HPV has three major oncoproteins E5, E6 and E7, that interact with the human immune system to downregulate the major histocompatibility complex (MHC) Class I, p53 and Rb respectively. Although infections are usually cleared by the immune system in a timely manner, albeit after a delay period, the virus can evade the immune system to persist and cause progression to a malignant disease under appropriate environmental conditions^10^.

HR-HPV types are regarded as the most important etiological factor for 95% of cervical cancer cases^11^. HPVs have also been found to be the related with the cause of a significant fraction of a variety of other cancers including vaginal, penile, anal, and oropharynx cancers^12,13^. These findings could indicate that HPV might be transported from the original infection site to other organs and may be responsible for the development of cancer in various organs. Indeed, our published findings have shown evidence for the presence of 10 HR-HPVs other than 16 and 18 in freshly obtained human breast cancer tissue which provides a solid basis to advance research in a crucial health imperative affecting women^14^.

The relationship between HPV and PCa is considered to be valuable for several reasons. Ravich and Ravich, first proposed in 1951 that sexually transmitted diseases (STDs) may also contribute to prostate carcinogenesis^15^. Since then, there have been several epidemiological studies focusing on the association between STDs and PCa^16–20^. STDs are generally known to increase the risk of PCa by causing inflammation of the prostate which may then lead to the initiation of carcinogenesis^4,21^. In 2019, it has been reported by Vignera et al. that 82% of patients with prostatitis have constant HR-HPV DNA in semen^22^.

Results of studies conducted thus far remain highly debatable, lacking concrete evidence for the presence of HPV in PCa^23–26^. Considering the broad interest in the association of HR-HPV types and PCa, it is considered to be very important to verify the prevalence of the various HR-HPV types, not just 16 and 18, in prostate tissues worldwide.

Despite the medical importance and the high incidence rate of PCa among the global population and the role of HR-HPV as a possible risk factor in the development of PCa no studies in the UK, to our knowledge, have been carried out on the role of HPV infection in the carcinogenesis of PCa. Hence, we aimed to examine the presence and expression of 12 HR-HPV types in the biopsies of benign and malignant fresh prostate tissue from patients from a UK population using highly sensitive molecular analysis.

## Results

### The identification of 12 high-risk HPV type sequences using PCR and Sanger sequencing

The pathological diagnosis results of the prostate tissue biopsies from patients with suspected prostate cancer are presented in Table 1. The pathological status presented was determined by the histopathology department of Kingston Hospital after biopsy, taking all biopsy material into account. Of the 49 samples (all from different patients) analyzed, 14 (29%) were classified as adenocarcinoma. Also 35 (71%) cases were classified as benign, where 23 (47%) benign prostate samples displayed signs of prostatic inflammation at pathological diagnosis.

**Table 1:** Pathological stratification and prevalence of high-risk HPV DNA in prostate samples (hyperplasia, benign and cancerous).

Age (Year) (56–92)	Total samples N (%)	Pathological status			HPV positive samples N (%)
		Benign	Benign with inflammation	Adenocarcinoma	
51–60	2 (4.1)	–	2	–	1/16 (6.3)
61–70	12 (24.5)	2	7	3	6/16 (37.5)
71–80	26 (53)	9	10	7	5/16 (31.3)
81–90	8 (16.3)	1	3	4	4/16 (25)
91–100	1 (2)	–	1	–	–
Total	49 (100)	12	23	14	16/49 (32.7)

The table demonstrates the HR-HPV prevalence of each age group. The age group with the highest HPV infection rate was 61–70 years. See Tables 2 and 3 for detailed result breakdown. Fisher’s exact test shows a significant association between age group and HPV status (*p* value 0.03462). Specifically, the odds of being HPV positive are 6.93 times higher in the 71–80 age group compared to other age groups. However, a wide 95% confidence interval of the odds ratio [1.16, 57.13] suggests that the small sample size may limit the precision of the odds ratio.

### PCR results

**Figure 1:**
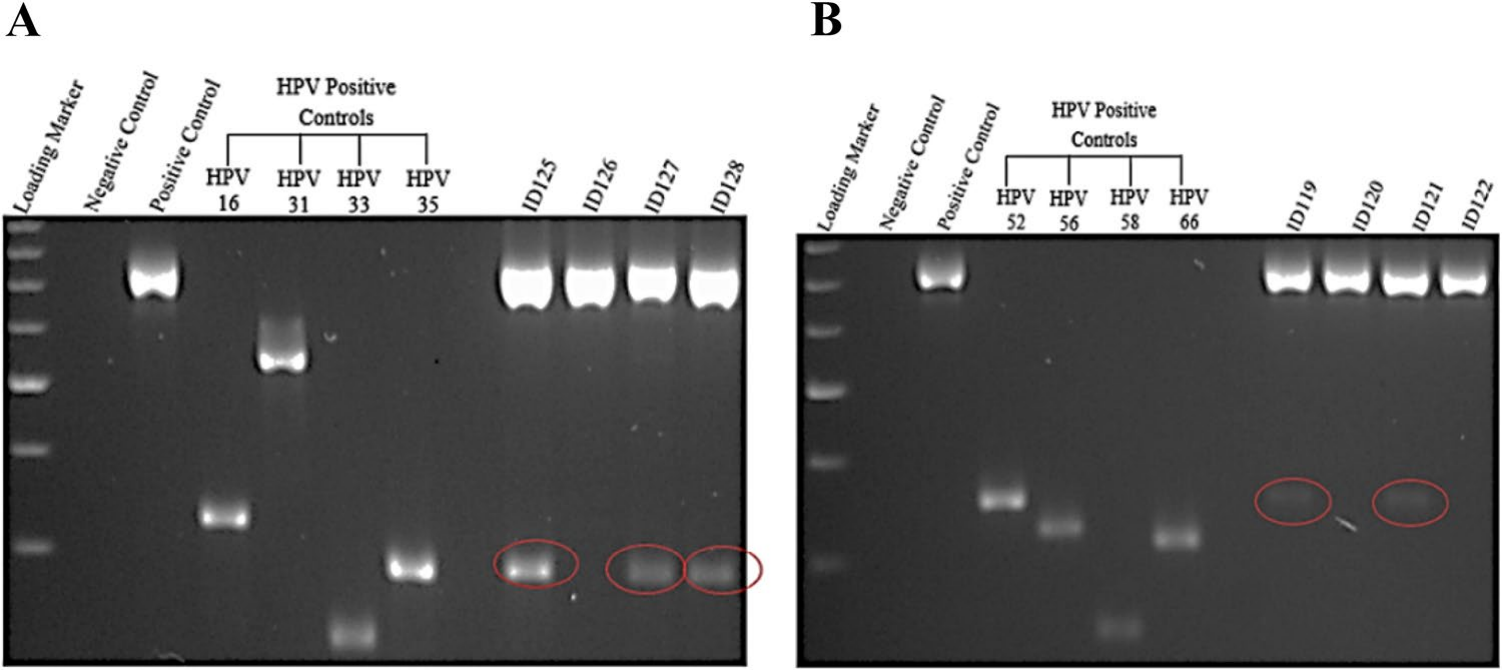
A representative gel electrophoresis pattern of 12 high risk HPV types by genotype specific primers amplification. (**A**) Gel electrophoresis pattern of high risk HPV types (16, 31, 33 and 35), Loading Marker = DNA ladder 100 bp plus (100 bp–3000 bp), HPV 16/35 = Positive Control DNA HPV types, 16 (325 bp), 31 (520 bp) and 33 (227 bp), 35 (280 bp) respectively, ID 126 = HPV Negative clinical sample, ID 125, 127 and 128 = HPV Positive clinical sample, Positive Control (PC+) = Internal control; human DNA (β-globin 723 bp). (**B**) Gel electrophoresis pattern of high-risk HPV types (52, 56, 58 and 66), HPV 52/66 = Positive Control DNA, HPV types 52 (360 bp), 56 (325 bp), 58 (240 bp) and 66 (304 bp) respectively, ID 120 and 122 = HPV Negative clinical sample, ID 119 and 121 = HPV Positive clinical sample.

A total of 49 fresh abnormal prostate tissue specimens (benign and malignant) from male patients aged between 56 and 92 years old were examined for the presence of 12 high-risk HPV DNA using HPV type specific PCR (Table 2). For each sample, the PCR experiment was repeated in triplicate to validate the accuracy of the obtained data. The amplification of the β-globin gene (fragment size 723 bp) was positive for all samples analyzed, indicating adequate quality of all samples. As a standard protocol, all HPV positive controls and negative controls were included for every sample run in gel electrophoresis. As shown in Fig. 1, the fragments of HPV positive and negative controls do not display any signs of contamination, further indicating efficient PCR amplification. In each case, Fig. 1A,B indicate successful PCR amplification of each HPV set of abnormal prostate samples.

**Table 2:** Type distribution of high-risk HPV genotypes in cancerous and benign prostate tissues. The HPV positivity rate was 32.7% (18.4–49.6%, 95% bootstrap CI) using the bootstrapping assay for the table.

Pathological status	HPV genotype											
HPV + cancerous cases n = 5 5/14 (36%)	16	31	33	35	18	39	45	59	52	56	58	66
	2/5 (40)	–	–	2/5 (40)	–	1/5 (20)	–	–	1/5 (20)	2/5 (40)	1/5 (20)	–
HPV + benign cases n = 11 11/35 (31%)	16	31	33	35	18	39	45	59	52	56	58	66
Prostatitis 9/23 (39%)	1/9 (11)	–	3/9 (33)	3/9 (33)	–	–	1/9 (11)	–	4/9 (44)	1/9 (11)	1/9 (11)	–
No prostatitis 2/12 (17%)	–	–	1/2 (50)	2/2 (100)	–	–	–	–	–	–	–	–
Total prevalence of specific HPV genotype cases n =	1/11 (9)	–	4/11 (36)	5/11 (45)	–	–	1/11 (9)	–	4/11 (36)	1/11 (9)	1/11 (9)	–

Prevalence of 12 HR-HPV types in prostate tissue samples. The table demonstrates the prevalence of each of the 12 HR-HPV types in cancerous and non-cancerous prostate tissue. Most prevalent HPV types in cancerous cases were HPV16, 35 and 56 with 2 samples infected. The most prevalent HPV type in non-cancerous cases was HPV35 with prevalence a total of 5 samples infected followed by HPV33 and 52 with a total of 4 samples infected. The data was bootstrapped, and the observed HPV positivity rate was 32.7% (18.4–49.6%, 95% bootstrap CI).

### Sanger sequencing results

**Figure 2:**
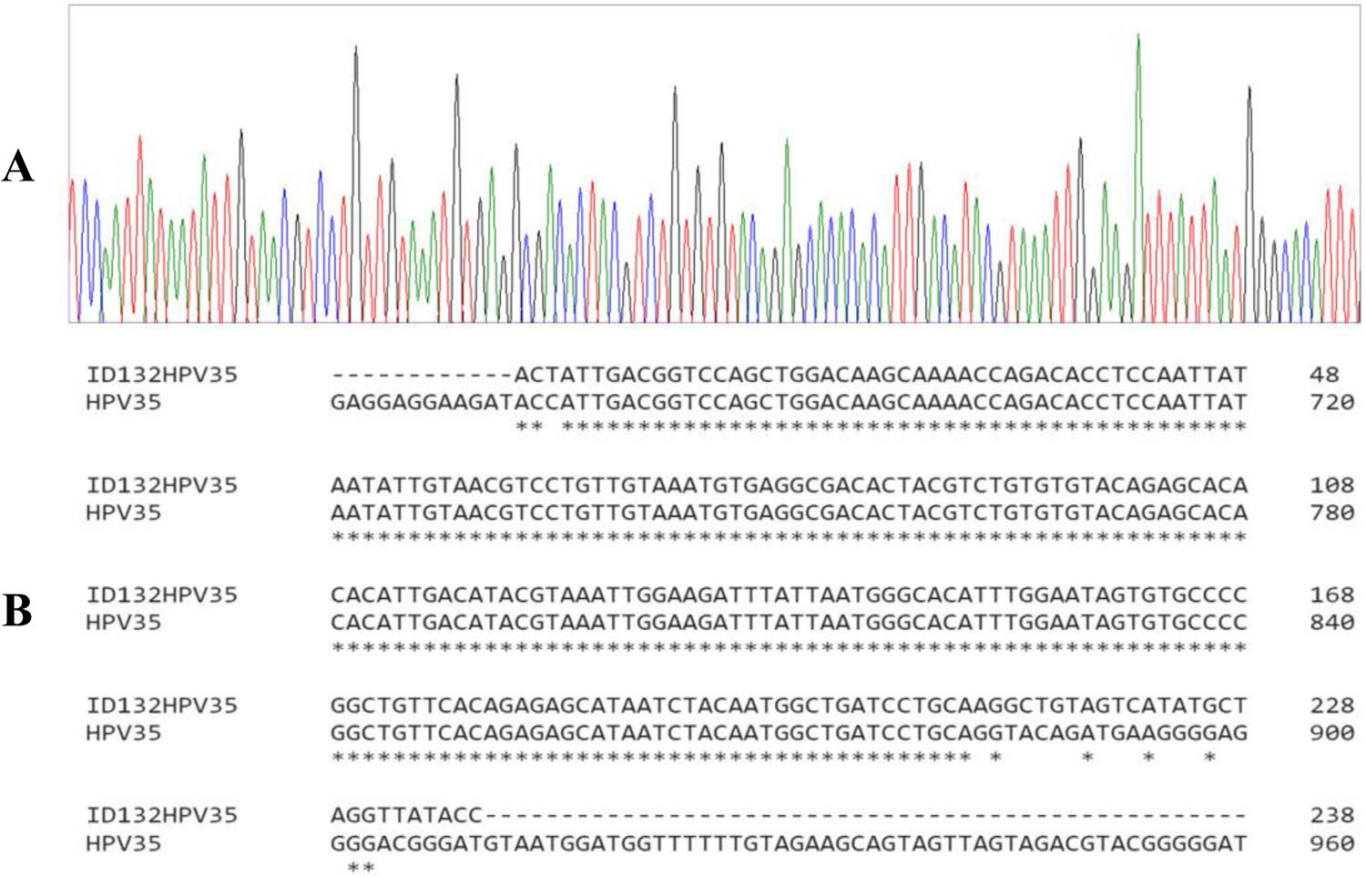
Sanger sequencing results further validate and confirm the PCR results. A representative Sanger sequencing result (Sample ID132) show > 90% concordance. This applies to all PCR results.

All high-risk HPV positive samples were subject to Sanger sequencing to validate the PCR results. Moreover, few HPV negative samples were also sequenced to serve as control. BLAST analysis revealed > 90% concordance in high-risk HPV positive amplified products. Data obtained from Sanger sequencing are consistent with PCR results which further validates our findings on the detection of HPV DNA. A representative of Sanger sequence analysis is shown in Fig. 2.

### Prevalence and type distribution of 12 HR-HPV types in prostate tissue

As shown in Tables 1, 2 and 3, the results demonstrate that HR-HPV DNA samples were detected in 16/49 (32.7%) of all benign and malignant prostate cases. In malignant cases, HR-HPV DNA was detected with a prevalence of 35.7% (5/14) (Table 2), in which, HPV types 16, 35 and 56 were the most prevalent HPV genotype with a prevalence of 40% (2/5) followed by HPV39 and 52 with prevalence of 20% (1/5) (Table 2). Moreover, in benign cases HPV was detected in 11/35 (31.4%) (Table 3) and the most prevalent HPV type in these cases was HPV35 with prevalence of 45.5% (5/11) followed by HPV33 and 52 with a prevalence of 44% (4/9), and HPV16, 45, 56 and 58 with a prevalence of 50% (1/2) (Tables 2, 3).

**Table 3:** HPV frequency of high-risk HPV infection, and co-infection with multiple high-risk HPV genotypes in cancerous and benign prostate tissue samples.

Pathological status	Total number of cases	Total HPV + N (%)	HPV co-infections N (%)
Cancerous cases	14/14 (100)	5/14 (35.7)	2/14 (14)
Adenocarcinoma	14	5/14 (35.7)	2/14 (14)
Benign cases	35/35 (100)	11/35 (31.4)	5/35 (14)
Benign (+ Prostatitis)	23	9/23 (39)	4/23 (17)
Benign (− Prostatitis)	12	2/12 (16)	1/12 (8)
Total	49	16	7

HR-HPV infections and co-infection with multiple HR-HPV genotypes in benign prostate tissues reveal that 39% of benign prostate samples with prostatitis had at least one HR-HPV genotype infection. HR-HPV coinfection was also observed to be 17% amongst the benign prostate samples with prostatitis. Fisher’s exact test indicates that there is no strong association between HR-HPV and pathological status due to the small sample size (p = 0.06349) (odds ratio = 1.246268, 95% CI = 0.2825222–6.0572126). However, it appears that the HR-HPV positivity rate in cancerous cases/adenocarcinoma and benign with prostatitis are higher than the HR-HPV positivity rate in benign without prostatitis group.

### The expression of HPV E7

Forty-nine benign and malignant prostate specimens were used for assessing HPV E7 oncoprotein expression. HPV E7 oncoprotein was clearly expressed in 16 HPV DNA positive samples (100%). There was no HPV E7 oncoprotein expression in all thirty-three samples negative for HPV DNA. These data representatives are illustrated in Table 3. These results were verified by a pathologist at Kingston Hospital. Interestingly, co-infected samples showed more intense staining for HPV E7.

All prostate samples with one HPV infection showed staining for HPV E7 expression using Immunohistochemistry. Additionally, all samples with multiple HPV infections showed darker staining for HPV E7 expression. These results were perfectly concordant to the HPV infections.

## Discussion

HPV infection is one of the most common sexually transmitted diseases (STDs) as a vast majority of sexually active individuals may be infected by HPV at some point during their lifetime. Globally, PCa is ranked as the second most common diagnosed malignancy after lung cancer in men. Data have suggested a possible association between the exposure to STDs, such as HPV, and increased rate of PCas^27^. Several studies investigated the link between HR-HPV and PCa, however, findings from these studies have not substantiated a firm link yet and generated considerable controversy. Although HR-HPVs are found to be present in prostate tissue, the viral load is relatively low compared to the high HR-HPV copy numbers in cervical cancer. This may suggest a slightly different role of HR-HPV in cancer development in different tissue types^28^.

Data from a meta-analysis included studies which showed a significant pathogenetic association between HPV infection and increased risk of PCa with HPV 16 being the most prevalent type^27,29–31^. Conversely, others revealed no evident link between HR-HPV and PCa^32–34^, hence considering HPV as an etiological factor for PCa still remains controversial. A number of studies have been conducted in 15 different countries to evaluate the presence of HR-HPV in prostate tissues. In these studies, HPV types 16 and 18 were the most common high-risk types isolated from PCa patients^28^. However, other reports have identified the presence of HR-HPV types, 31, 33, 45, 52 and 58 in benign and malignant prostate tissues^35–37^. In this study we have examined the association of 12 HR-HPV types in the abnormal prostate specimens and found HPV 35 to be the most prevalent HR-HPV type in benign and malignant prostate tissues with a prevalence of 45.5% and 40% respectively (18.4–49.6%, 95% bootstrap CI) (Table 2, Fig. 2). HPV35 was also observed to be the most common HR-HPV type to be part of a HR-HPV coinfection. In contrast to previous studies, HPV 18 was not detected in any of the prostate specimens examined. Unlike our study, a number of reports have identified HPV types 16 and 18 in prostate samples, but these relied on screening a limited number of HR-HPV types (16 and 18 only). This in turn limited the detection of many other HR HPV genotypes that have been detected in this study. The absence of HPV type 18 and presence of other HR-HPVs in this population suggest that HR-HPV types vary within populations and geographical locations. Additionally, a role of HPV vaccination against HPV type 16 and 18 may be a reason for the absence of type 18. Indeed, study by Mesher et al. (2016) provides clear evidence of a reduction in the HPV vaccine types in the population with the highest HPV vaccination coverage in England^29,37,38^. Our research findings on the association of HPV and breast cancer have also established a similar trend of reporting differences in the HR-HPV type distributions among two different populations in two different geographic regions (the UK and Qatar)^14,39^.

Furthermore, findings from different age groups show that the highest percentage of HPV positive cases were recorded in two sets of populations aged 61–70 years and 71–80 years with a prevalence of 37.5% and 31.3% respectively (Table.1). These were found to be statistically significant using Fisher’s exact test. The odds ratio of being HPV positive are lower in the younger age groups, compared to the older age groups (*p* < 0.05). Therefore, the result is significant at *p* < 0.05. Conceivably, higher HPV infection rates amongst the older population may be a result of a weakened immune system and/or accumulation of other health related factors, which may aid viral persistence and progression from infectious stage to carcinogenesis.

**Figure 3:**
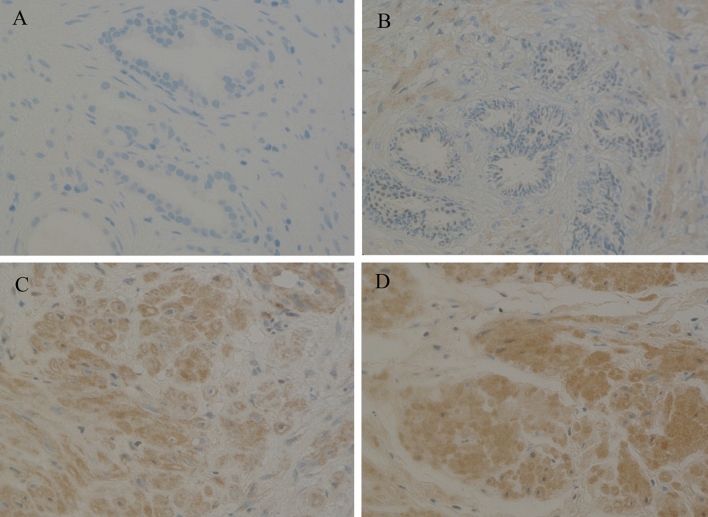
Expression of HPV oncoprotein E7 analyzed using Immunohistochemistry. (**A**) Representative of Prostate tissue with negative HPV expression. (**B**, **C**) Representative of benign and malignant prostate samples, respectively with positive HPV expression and (**D**) representative of HPV co-infected prostate tissue with more intense. HPV expression. Images were analyzed using ImageJ IHC analyzer plug-in.

Comparing the HPV positivity rate with the pathological status showed no statistically significant differences (Table 3) *p* value 0.06349. However, analysis of HPV protein expression showed that if more than one HPV type is present in the prostate tissue, the HPV oncoprotein infection is more active. 50% of the co-infected benign prostatic tissues with inflammation showed darker staining for HPV protein expression. Likewise, the only co-infected benign tissue without inflammation also displayed strong positive HPV protein expression (Fig. 3). There may be a possibility that the non-inflamed prostate tissue infected with multiple HR-HPV types may subsequently develop inflammation, aiding the virus to persist and probably induce prostate cancer. Additionally, analyzing the IHC, all HPV DNA positive samples showed the expression of the HPV protein, which could indicate that the prostate tissue may provide the optimum conditions for the virus to persist and progress to cancer. Then again, further research is required to confirm this. This data is the first to establish the activity of a co-infection in the prostate tissue, which is not limited to HPV16 and HPV18 infection.

Glenn et al.^35^, have anticipated that the presence of HR-HPV in benign prostate tissue may trigger the development of PCa about a decade later. This suggests that HR-HPV may remain biologically active for several years before the development of PCa, and not just be a harmless passenger virus of the prostate tissue. Also, the immune response decreases significantly as men age which may lead to immunodepression and accumulation of other infections which provides ideal conditions for HR-HPV to thrive in.

Similarly, Medel-Flores et al.^36^ suggested that prostatitis might be caused by HR-HPVs which may prompt PCa development. We have also observed similar trends in our study as patients with prostatitis presented with a higher number of HPV positive cases and co-infections with a frequency of 39%, with 17% of the HPV infected samples being co-infected. This may imply that the inflammation of the prostate may aid the virus in the development of cancer, yet further studies are required to confirm this.

It has also been reported that inflamed prostate tissues were associated with multiple genotypes in a single sample (co-infection)^28^. Likewise, the present study shows an occurrence of HPV co-infection in 14% of HPV positive invasive PCa cases, however, these were not found to be significant odds ratio = 1.246268, 95% CI = 0.2825222–6.0572126 (Tables 3, 4). Moreover, this study establishes that the most prevalent HPV types in cancerous cases were HPV 16, 35 and 56 with a prevalence of 40% followed by HPV 39 and 52 with prevalence of 20%. On the other hand, HPV 35 was the most frequent type in non-cancerous cases with prevalence of 45.5% followed by HPV33 and HPV52 with a prevalence of 36.4%, HPV16, 45, 56 and 58 with a prevalence of 9.1% respectively. Our results are in accordance with previous reports that suggested a probable association between HPV and risk of inflammation in prostate tissue, which may both contribute to the risk of developing PCa.

**Table 4:** HPV coinfection combination types in prostate tissues.

HPV co-infection pairing	Frequency
HPV16-HPV56	1
HPV33-HPV35	2
HPV33-HPV52	1
HPV16-HPV35-HPV56	1
HPV16-HPV39-HPV56	1
HPV35-HPV52-HPV58	1

HPV coinfections show that the HPV33–HPV35 coinfection combination was most observed. Moreover, HPV16 and HPV56 were involved in the most coinfections along with another HPV type. This was observed in 3/7 coinfection cases.

There have been several studies conducted in the past that have used a similar approach to determine the activity of HPV in prostate samples. However, these studies were limited to only two HR-HPV types (HPV 16 and HPV 18). We have identified multiple HR-HPV types that are present in fresh prostate tissue. Moreover, according to our knowledge this is the first such study to be conducted within the UK population. The detection of viral DNA indicated the presence of HPV, but not a productive infection. Therefore, IHC was carried out to determine whether the HPV protein was expressed in samples positive for HPV DNA**.** Indeed, data obtained from this investigation confirms that the HPV protein is actively expressed in all HPV DNA positive tissues. These findings are parallel to the trend observed by Ngan et al. (2015) in breast cancer, indicating there may be a role for HPV in the onset of prostate cancer, however, this may need long term observation (1–11 years) and regular follow ups should be necessary^40^.

Due to the asymptomatic nature of PCa until the later stages of the disease, it is considered important to identify the potential etiological factors linked to these lesions in an effort to implement prophylactic measures to help prevent their effects and improve patient quality of life. Although the current vaccine is fairly successful, additional HR-HPVs have been identified in this study that the vaccine does not protect against^41^. Even if an association of HPV with PCa development is proven, given the other risk factors known to influence the risk of PCa such as age, infection, testosterone and ethnicity, the possibility of overall benefit of implementing the current vaccine alone to reduce the risk of developing PCa could be small. Therefore, public awareness against the impact of HPV and the development of vaccination against a broader range of HR-HPV subtypes may be required in the future, if further studies prove the involvement of different HR-HPV types in prostate cancer development.

There are some limitations to this study. The small sample size is probably the biggest limitation. However, the data obtained from this sample size provide evidence of high-risk HPV presence in prostate tissues. This finding can be explored further using statistical analysis using larger sample size in the future to determine whether high-risk HPVs have a significant part in PCa progression. Similarly, due to the small sample size it is difficult to draw meaningful comparative statistical analysis and conclusion of prostatitis versus non-prostatitis tissues. Finally, it would be of great interest to evaluate the statistical differences between the presence of HPV in prostate cancer patients and general population. However, HPV prevalence is different amongst populations in different geographical locations. No such study has been conducted in UK prior to this, therefore the HPV prevalence from another population would not be achievable.

## Materials and methods

### Recruitment of patients and prostate tissue specimen collection

The study protocol was conducted in accordance with the Declaration of Helsinki and officially approved by the Ethics Committee of Health and Research Authority NHS (NRES Committee East Midlands – Leicester Central, UK) REC reference: 17/EM/0393. All methods were carried out in accordance with the approved guidelines and regulations. Following informed consent, 49 fresh prostate specimens (benign and cancerous) were collected aseptically by one surgical team (over a period of 2 years) and preserved immediately using AllProtect reagent (Qiagen, Hilden, Germany) to stabilize the DNA, RNA and Proteins. All specimens were reported at Kingston Hospital Histopathology department, London, UK.

### DNA extraction and purification

To avoid cross-contamination between specimens during tissue handling, tissues were handled aseptically using disposable items, such as gloves, surgical blades and tubes. Cellular DNA, RNA and protein were extracted from the collected prostate tissue samples using GenElute RNA/DNA/Protein Purification Plus kit (Sigma-Aldrich) in accordance with the manufacturer’s protocol. Briefly, to achieve purified genomic materials and protein, subsequent steps were followed: prostate tissue was accurately measured with a weight ranging between 20 and 30 mg and then homogenized in lysis buffer using Tissue Lyser® (Qiagen, Hilden, Germany) and QIAshredder spin columns (Qiagen, Hilden, Germany). The lysate of each sample was loaded into a gDNA purification column to bind the DNA. The genomic material was washed and then eluted using allocated elution buffer as instructed by the manufacturer in order to obtain the purified nucleic acid. The concentration and purity of the extracted nucleic acids were assessed using NanoVue plus spectrometer (GE Lifesciences, Chicago, Illinois, USA).

### Detection and genotyping of HPV DNA

To identify the 12 different HR-HPV subtypes in prostate tissues, the selected research approach was Polymerase Chain reaction (PCR). This was achieved using the HPV-HCR Genotype-Eph kit (AmpliSens, Bratislava, Slovak Republic) along with the purified DNA extracts of prostate tissues. In order to avoid cross-contamination, DNA extraction and PCR technique were performed at two separate laboratories under a very strict aseptic protocol. Simultaneous amplification of four targeted regions of the HPV gene of four different HPV DNA types was run in one tube (multiplex-PCR). The HPV DNA amplification was carried out in three tubes, with each tube distinctly amplifying the following HPV types: 16/31/33/35, 18/39/45/59 and 52/56/58/66. This approach allowed the identification of HPV infections and co-infections of 12 distinct high-risk HPV subtypes in each sample. For each sample, the PCR experiment was repeated in triplicate to validate the accuracy of the obtained data. The amplification of the β-globin gene (fragment size 723 bp) was positive for all samples analyzed, indicating adequate quality of all samples.

To analyze and determine HPV genotypes, the amplified PCR products were electrophoresed along with type specific positive HPV controls on a 3% (w/v) agarose gel stained using SYBR Safe (Invitrogen, Carlsbad, CA, USA). For each sample, β-globin amplification served as an internal control to determine the adequacy of the extracted DNA for amplification. This was then visualized under UV using a Gel Doc XR + System (Bio-Rad, Hercules, CA, USA).

### HPV DNA sequencing

In order to confirm the presence of HPV genes, amplified PCR products of the HPV positive samples were subjected to direct sequencing. All PCR products were purified using a QIAquick PCR Purification Kit (Qiagen, Hilden, Germany) and then sequenced using an Applied Biosystems 3730xL analyzer. The results were analyzed using NCBI BLAST program to verify HPV sequences. The use of this method was used to further validate the results.

### Statistical analysis

The prevalence of HPV was calculated as the number of HPV-positive samples divided by the total number of samples tested, whereas the genotype-specific prevalence was calculated as the number of samples positive for a given genotype with or without co-infection with other genotypes divided by the number of all samples tested. A Fisher's exact test was performed using R software to determine whether there was a significant association between HPV status and pathological status. The analysis involved creating a 2 × 5 contingency table with rows representing HPV positive and HPV negative samples, and columns representing the five age groups: 51–60, 61–70, 71–80, 81–90, 91–100. The contingency table was then used to calculate the p-value, odds ratio, and 95% confidence interval for the test. A significance level of 0.05 was used for the test. Similarly, Fisher’s exact test also involved creating a 2 × 3 contingency table with rows representing HPV positive and HPV negative samples, and columns representing the three pathological status groups: cancerous, benign prostatitis, and benign non-prostatitis. The contingency table was then used to calculate the p-value, odds ratio, and 95% confidence interval for the test. A significance level of 0.05 was used for the test.

Bootstrapping confidence interval for genotype specific prevalence for prostate samples. Analysis were performed by using R software (version 4.2.2) and boot package for bootstrapping. The level of statistical significance was set at 0.05.

### Immunohistochemistry

In order to confirm the expression of HPV genes, HPV positive samples were subjected to Immunohistochemistry using anti-HPV E7 monoclonal antibody (Cervimax) – Valdospan GmbH, Austria, which reacts with a wide range of High-Risk HPV types. Standard automated VENTANA automated immunohistochemistry protocol by BenchMark Ultra (Roche) was applied. The HPV E7 monoclonal antibody was used at a 1–100 dilution for 1 h at 36 °C as per manufacturer protocol. Cervical Intraepithelial Neoplasia grade three (CIN III) blocks were used as positive control and slides incubated in serum without HPV E7 antibody were used as negative control.

IHC staining was then examined using ImageJ ‘IHC analyzer’ Plug in. The intensity of the stain was calculated by ImageJ in pixels and was scored as negative; no staining, positive; only visible at high magnification, strongly positive; visible at low magnification.

## Conclusions

This study investigated the presence of 12 HR-HPV DNA in fresh prostate tissue samples from a population of UK patients for the first time wherein viral DNA was detected in 35.7% of PCa cases. This suggests that HR-HPVs may play a role in PCa. This is further supported by the data collected with IHC, where all HR-HPV infected samples display positive and strong positive HPV expression.

To date, PCa was not evidently linked to any preventable risk factors such as HR-HPV infection. However, our findings show that HR-HPV is present in prostate tissue. It may remain biologically active for several years before the development of PCa. However, further research is required to confirm the actual role of HPV in Prostate cancer.

## Data Availability

The datasets generated and/or analysed during the current study are available in the GenBank repository [Accession number BankIt2645736 OP863213].
